# Evaluation of the Safety Profile of Direct-Acting Antivirals on Patients with Hepatitis C Virus: A Pharmacovigilance Study

**DOI:** 10.1007/s43441-023-00537-x

**Published:** 2023-05-25

**Authors:** Mai G. El-Marakby, Mohamed H. Solayman, Nagwa A. Sabri

**Affiliations:** Clinical Pharmacy Department, Faculty of Pharmacy, Ain Shams University, Cairo, Egypt

**Keywords:** Direct-acting antiviral, Hepatitis C virus, Pharmacovigilance, Adverse reaction, VigiBase

## Abstract

**Background:**

Hepatitis C virus (HCV) is the primary contributor to chronic hepatic diseases. A rapid change in the situation took place with the advent of oral direct-acting antivirals (DAAs). However, a comprehensive review of the adverse event (AE) profile of the DAAs is lacking. This cross-sectional study aimed to analyze the reported Adverse Drug Reactions (ADRs) with DAA treatment using data from VigiBase, the WHO Individual Case Safety Report (ICSR) database.

**Methods:**

All ICSRs reported to VigiBase with sofosbuvir (SOF), daclatasvir (DCV), sofosbuvir /ledipasvir (SOF/LDV) and ombitasvir/paritaprevir/ritonavir (OBV/PTV/r) in Egypt were extracted. Descriptive analysis was performed to summarize patients’ and reactions’ characteristics. Information components (ICs) and proportional reporting ratios (PRRs) for all reported ADRs were calculated to identify signals of disproportionate reporting. Logistic regression analysis was performed to identify the DAAs association with serious events of concern while adjusting for age, gender, pre-existing cirrhosis, and ribavirin use.

**Results:**

Out of 2925 reports, 1131 (38.6%) were serious. The most commonly reported reactions; anaemia (21.3%), HCV relapse (14.5%) and headache (14%). For the disproportionality signals; HCV relapse was reported with SOF/DCV (IC 3.65, 95% CrI 3.47–3.79) and SOF/RBV (IC 3.69, 95% CrI 3.37–3.92), while anaemia (IC 2.85, 95% CrI 2.26–3.27) and renal impairment (IC 2.12, 95% CrI 0.7–3.03) were reported with OBV/PTV/r.

**Conclusion:**

The highest severity index and seriousness were reported with SOF/RBV regimen. A significant association was found for OBV/PTV/r with renal impairment and anaemia although being the superior regimen in terms of efficacy. The study findings call for further population-based studies for clinical validation.

## Introduction:

Hepatitis C virus (HCV) infection is a major cause of morbidity and mortality [1]. About 58 million people worldwide are diagnosed with hepatitis C and nearly 1.5 million infections occur per year [2, 3]. Approximately 290,000 people died from hepatitis C in 2019 [2].

Egypt has one of the highest global burdens of hepatitis C virus (HCV) infection [4]. High rates of HCV transmission in Egypt were associated with the nationwide parenteral anti-schistosomal therapy campaigns from the 1950s to the 1980s, due to a series of unsafe intravenous injections [1, 5].

The estimated prevalence in the Egyptian population was 14.7% in 2008 [1, 6], dropped to 10% in 2015 [1, 7] then dropped further to 4.6 in 2019 [4, 6, 7]. Hepatitis C virus is the primary contributor to chronic liver diseases in Egypt, including cirrhosis and hepatocellular carcinoma (HCC) [8], those comorbidities has been ranked by the WHO among Egypt’s top 10 causes of death [9–11].

However, a rapid change in the situation took place in Egypt [11] after the conduction of multiple national programs to screen and treat HCV. A lower rate of HCV prevalence of 4.6% was reported in a large-scale national study conducted during 2018–2019 [4, 7].

The Historic treatment of HCV was the combination of pegylated interferon and ribavirin, but this was associated with poor virological response in addition to poor safety and tolerability [1, 12]. Chronic HCV treatment outcome was dramatically altered by the introduction of the oral direct-acting antiviral (DAA) drugs [1]; sofosbuvir (SOF), daclatasvir (DCV), sofosbuvir/ledipasvir (SOF/LDV), and ombitasvir/paritaprevir/ritonavir (OBV/PTV/r) [1, 13]. DAAs offer a short‐term treatment duration with high rates of sustained virologic response (SVR) [4].

Few serious adverse drug reactions (ADRs) associated with DAAs were observed and reported in the clinical trials performed on the DAAs, however, clinical trials are limited in their ability to detect ADRs that occur in a ‘real-world’ setting and a broad review of the adverse event (AE) profile with DAAs is lacking [14]. Therefore, post-marketing surveillance through pharmacovigilance activities-as spontaneous reporting of ADRs- is essential to identify potential safety and efficacy concerns [13, 15].

Although over five million patients were treated with DAAs globally [13] and more than two million patients were treated in Egypt by 2018 [7], there are no large data-based studies with an extended period were conducted in Egypt to detect potential associations between reported adverse events and DAA drugs.

This pharmacovigilance study using VigiBase—the World Health Organization global database of individual case safety reports (ICSRs)—aims to evaluate the safety profile of the direct-acting antivirals (DAAs) regimens (SOF + RBV, SOF + DCV, SOF/LDV, and OBV/PTV/r) on the local population in Egypt through describing the pattern of adverse events, and detecting the signals of disproportionate reporting (SDRs)—statistical associations between medicinal products and adverse events- [16, 17].

## Materials and Methods

### Study Design and Data Source

This is a cross-sectional pharmacovigilance study conducted using data from VigiBase—developed and maintained by Uppsala Monitoring Centre (UMC)- which contains about 29,699,362 reports received from around 140 countries. VigiBase receives reports of ADRs occurred with medicinal products from the national centres for pharmacovigilance in the participating countries [18, 19].

The information in VigiBase comes from different sources and the probability that the suspected adverse effect is drug-related is not the same in all cases. Study results and conclusions do not represent the opinion of the UMC or the World Health Organization [20].

### Ethics Approval

The data used in the study were anonymized and there was no direct interaction with human participants. Approval to conduct this study and extract data from VigiBase was obtained from the Egyptian Drug Authority (EDA). The study protocol and methodology were revised and approved by the Committee of Ethics, Faculty of Pharmacy, Ain Shams University (approval number: 236 dated 13-03-2019).

### Data Extraction and Analysis

For this study, all ICSRs with designated direct-acting antivirals (DAAs) reported in Egypt since the introduction of DAA regimens in 2013 till the 7th of September 2021 (data extraction date) were selected. All reports with the following suspected drugs were included: sofosbuvir (SOF), daclatasvir (DCV), sofobuvir/ledipasvir (SOF/LDV), and ombitasvir/paritaprevir/ritonavir (OBV/PTV/r) for the treatment of Hepatitis C Virus (HCV).

The data sets were extracted from VigiBase to Microsoft Excel files using the standard terminology in VigiBase as follows; Anatomical Therapeutic Chemical (ATC) classification of medications, the active substance of the medicines, according to WHO Drug dictionary for medicinal products and the Preferred Term (PT) level in the Medical Dictionary for Regulatory Activities (MedDRA) for coding the ADRs.

The extracted data were then reviewed to exclude reports with concomitant interferon (IFN), and reports that included SOF alone or DCV alone as they are not approved as monotherapies. Moreover, a case-by-case review was performed to obtain additional data pertaining to the severity assessment of the ADR and the logistic regression analysis.

The reports were then analysed based on age, gender, causality, System Organ Class (SOC) and Preferred Term (PT) levels of the ADR, ADR seriousness and type of seriousness, and ADR severity.

Severity assessment of the ADR outcome was performed and classified utilizing Hartwig's severity assessment scale, which is divided into seven levels [21–24].

Any ADR that was associated with death, life-threatening condition, hospital admission or prolongation of hospitalization, persistent disability, congenital anomaly and any medically important event or condition that was considered clinically critical by the reporting health care professional, is considered a serious reaction according to the ICH definition [25].

Causality was defined as probability that the drug caused the adverse event [26]; which differs between case reports [20], so causality was assessed and categorized by the reporter according to WHO-UMC criteria [22, 27].

### Statistical Analysis

The statistical analysis was performed using R software (v. 4.1.1). *P*-values less than 0.05 were considered to represent statistically significant results [28].

### Descriptive Analysis

Descriptive statistics for patients’ characteristics, system affected (SOC), reaction PT, seriousness and type of seriousness, causality, and outcome severity all were presented as frequencies and percentages. Distributions of the aforementioned data were compared among regimens using the *χ*^2^ or Fisher’s exact tests.

### Disproportionality Analysis

Disproportionality analysis - which is a tool for signal identification by describing the extent to which the reported adverse drug reaction is related to the suspected drug compared with the other drugs in the database was performed [29].

Information components (ICs) and Proportional Reporting Ratios (PRRs) –which are indicator quantities used to describe the disproportionality in reporting- were calculated for each ADR per treatment regimen. PRRs greater than 2, ICs greater than 1, IC 95% credibility interval (95% CrI) lower-bound greater than 0, and PRR 95% confidence interval (95% CI) lower-bound greater than 1 were all considered as signals of disproportionate reporting (SDR). In addition, the minimum number of reports required to detect a signal was 3 [29–31].

### Logistic Regression Analysis

Logistic regression analyses were performed to calculate the reporting odds ratio (ROR) and investigate the association between the exposure to a DAA and the pattern of four specific serious events, while adjusting for the effect of covariates that possibly having an impact on the pattern of these ADRs including, age, gender, ribavirin use, and pre-existing cirrhosis. The four selected events were HCV/HBV reactivation, anaemia, renal impairment (including AKI, renal insufficiency, and elevated blood creatinine), and hepatic complications (composite endpoint including hepatocellular carcinoma and hepatic encephalopathy). The aforementioned events were selected as being serious and co-reported with the four DAAs of interest.

**Figure 1.**
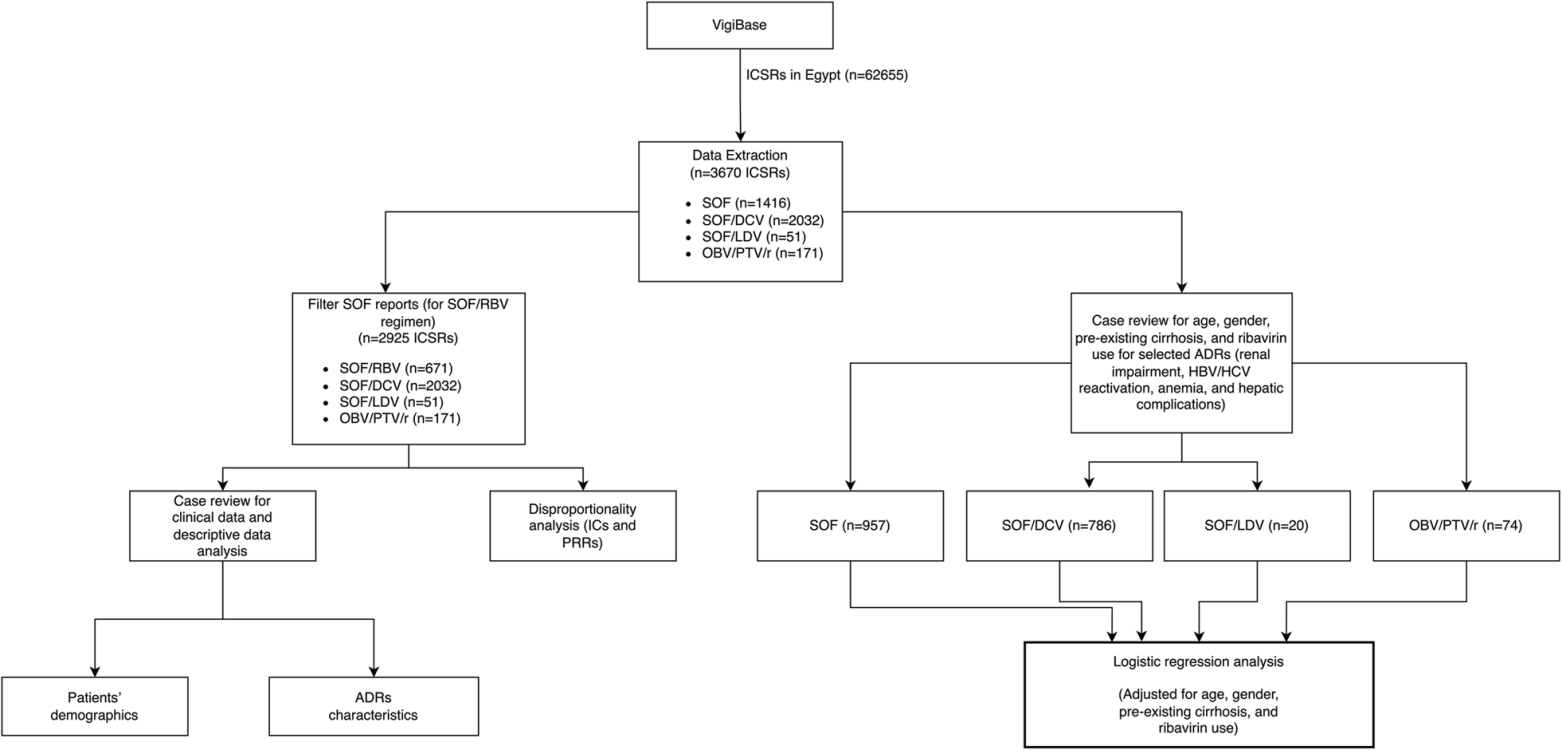
Study Methodology Flowchart. *ICSR* Individual Case Safety Report, *ADR* adverse drug reaction, *SOF* sofosbuvir, *DCV* daclatasvir, *LDV* ledipasvir, *OBV/PTV/r* ombitasvir/paritaprevir/ritonavir, *ICs* information component, *PRRs* proportional reporting ratios, *HCV* hepatitis C virus, *HBV* hepatitis B virus.

Preceding this analysis, case review was performed to include only reports with the aforementioned events and covariates. Reports with SOF only and SOF/IFN regimens *were not excluded* from the case review and logistic regression analysis and were merged with SOF/RBV reports under a reference group named ‘Legacy treatment’, denoting the HCV treatment regimens used before the release of newer DAAs including DCV and LDV, which were thereafter used in combination with SOF [2, 12]. The study flowchart is shown in Fig. 1.

## Results

The total number of reports from Egypt in VigiBase on the data extraction date was 62,655 reports. The total number of reports eligible for the descriptive and disproportionality analyses was 2925 reports with the DAA regimens of interest; sofosbuvir + ribavirin (*n* = 671), sofosbuvir + daclatasvir (*n* = 2032), ombitasvir/paritaprevir/ritonavir (*n* = 171) and sofosbuvir/ledipasvir (*n* = 51). 1385 (47.3%) of the patients aged 45–64 years and 1363 (46.6%) were females.

Moreover, 1131 (38.6%) of reports were serious ADRs (0.9% for death, 1.2% for life-threatening events, 1.6% for caused/prolonged hospitalization and 35.7% for other medically important condition). The percentage of fatal outcomes ranged from 0% with ombitasvir/paritaprevir/ritonavir to a maximal of 2% with sofosbuvir/ledipasvir. Besides, 1450 (49.5%) of ADRs were of mild severity (levels 1 and 2). The highest percentage of severe cases was reported with sofosbuvir/ribavirin (4.02%) while the lowest percentage was reported with ombitasvir/paritaprevir/ritonavir (0.53%). Concerning causality, 4932 (97.1%) of the ADRs reported have been assessed as possibly related to the suspected drug.

Among the reported ADRs, the most commonly involved organ system was the nervous system disorders (27.3%) with the highest percentage reported with sofosbuvir/daclatasvir (35.14%), blood and lymphatic system disorders (25.5%) with the highest percentage reported with sofosbuvir/ribavirin (59.5%), general disorders and administration site conditions (25.4%) with the highest percentage reported with ombitasvir/paritaprevir/ritonavir (40.9%), infections and infestations (16%) with the highest percentage reported with sofosbuvir/ribavirin (17.6%), skin and subcutaneous tissue disorders (7%) with the highest percentage reported with ombitasvir/paritaprevir/ritonavir (23.4%).

The most common ADRs according to PT level were anaemia (21.3%), hepatitis C relapse (14.5%), headache (14%), fatigue (12.9%), dizziness (12.2%) and pruritus (4.8%) (Table 1).

**Table 1:** Characteristics of Reports Extracted from VigiBase on DAAs for HCV Treatment.

	Active Ingredient				Total, *n* (%)	*P*
	SOF/RBV, *n* (%)	DCV/SOF, *n* (%)	SOF/LDV, *n* (%)	OBV/PTV/r, *n* (%)		
Gender						
Female	69 (10.28)	1202 (59.04)	14 (27.5)	78 (44.97)	1363 (46.6)	< 0.001^a^
Male	176 (26.23)	643 (31.83)	29 (56.9)	81 (46.03)	929 (31.76)	< 0.001^a^
Unknown	426 (63.49)	187 (9.23)	8 (15.7)	12 (8.99)	633 (21.64)	< 0.001^a^
Age group (year)						
2–11 years	0 (0)	0 (0)	14 (27.5)	0 (0)	14 (0.48)	< 0.001^b^
12–17 years	0 (0)	0 (0)	4 (7.8)	0 (0)	4 (0.14)	< 0.001^b^
18–44 years	33 (4.92)	553 (27.16)	4 (7.8)	27 (15.9)	617 (21.09)	< 0.001^b^
45–64 years	167 (24.89)	1102 (54.22)	12 (23.5)	104 (57.7)	1385 (47.35)	< 0.001^a^
65–74 years	17 (2.53)	196 (9.63)	3 (5.9)	30 (16.4)	246 (8.41)	< 0.001^b^
≥ 75 years	1 (0.15)	15 (0.74)	0 (0)	7 (3.7)	23 (0.79)	< 0.001^b^
Unknown	453 (67.51)	166 (8.25)	14 (27.45)	3 (6.3)	636 (21.74)	< 0.001^b^
ADR category						
Serious	575 (85.69)	526 (25.9)	18 (35.3)	12 (9.5)	1131 (38.67)	< 0.001^a^
Non serious	95 (14.16)	1495 (73.6)	33 (64.7)	156 (88.9)	1779 (60.82)	< 0.001^a^
Unknown	1 (0.15)	11 (0.5)	0 (0)	3 (1.6)	15 (0.51)	0.07^b^
Death	8 (1.19)	18 (0.88)	1 (2)	0 (0)	27 (0.92)	0.43^b^
Life threatening	18 (2.68)	16 (0.79)	1 (2)	1 (0.5)	36 (1.23)	< 0.01^b^
Caused/Prolonged hosp.	27 (4.02)	16 (0.79)	5 (9.8)	1 (1.6)	49 (1.68)	< 0.001^b^
Permanent disability	0 (0)	0 (0)	1 (2)	0 (0)	1 (0.03)	< 0.001^b^
Disabling/incapacitating	0 (0)	0 (0)	0 (0)	0 (0)	0 (0)	–
Other medically important condition	529 (78.84)	490 (24.1)	16 (31.4)	10 (8.5)	1045 (35.73)	< 0.001^a^
ADR severity						
Mild	49 (7.3)	1294 (63.68)	11 (21.57)	96 (50.79)	1450 (49.57)	< 0.001^a^
Moderate	440 (65.57)	304 (14.96)	5 (9.8)	15 (7.94)	764 (26.12)	< 0.001^a^
Severe	27 (4.02)	34 (1.67)	2 (3.92)	1 (0.53)	64 (2.19)	< 0.01^b^
Unknown (No data)	155 (23.1)	400 (19.69)	33 (64.71)	59 (31.22)	647 (22.12)	< 0.001^a^
System Organ Class						
Nervous system disorders	42 (6.26)	714 (35.14)	8 (15.69)	37 (21.64)	801 (27.38)	< 0.001^a^
Blood and lymphatic system disorders	399 (59.46)	306 (15.06)	7 (13.73)	34 (19.88)	746 (25.5)	< 0.001^a^
General disorders and administration site conditions	114 (16.99)	553 (27.21)	8 (15.69)	70 (40.94)	745 (25.47)	< 0.001^a^
Infections and infestations	118 (17.59)	343 (16.88)	7 (13.73)	2 (1.17)	470 (16.07)	< 0.001^b^
Skin and subcutaneous tissue disorders	18 (2.68)	144 (7.09)	3 (9.8)	40 (23.39)	205 (7.01)	< 0.001^b^
Preferred term						
Anaemia	385 (57.38)	202 (9.94)	5 (9.8)	32 (18.71)	624 (21.33)	< 0.001^a^
Hepatitis C relapse	109 (16.24)	308 (15.16)	6 (11.7)	2 (1.17)	425 (14.53)	< 0.001^b^
Headache	17 (2.53)	375 (18.45)	3 (5.88)	15 (8.77)	410 (14.02)	< 0.001^b^
Fatigue	34 (5.07)	309 (15.21)	3 (5.88)	34 (19.88)	380 (12.99)	< 0.001^b^
Dizziness	7 (1.04)	335 (16.49)	1 (3.92)	15 (8.77)	358 (12.24)	< 0.001^b^
Pruritus	6 (0.89)	99 (4.87)	2 (3.92)	35 (20.47)	142 (4.85)	< 0.001^b^
ADR causality						
Certain	8 (0.96)	1 (0.03)	0 (0)	0 (0)	9 (0.18)	< 0.001^b^
Probable	8 (0.96)	20 (0.53)	0 (0)	7 (1.9)	35 (0.69)	0.01^b^
Possible	804 (96.06)	3717 (98.28)	83 (93.26)	328 (89.13)	4932 (97.16)	< 0.001^a^
Unlikely	13 (1.55)	28 (0.74)	5 (5.62)	31 (8.42)	77 (1.52)	< 0.001^a^
Conditional	0 (0)	4 (0.11)	1 (1.12)	2 (0.54)	7 (0.14)	< 0.01^b^
Unassessable	4 (0.48)	12 (0.32)	0 (0)	0 (0)	16 (0.32)	0.54^b^

*DAA* direct-acting antiretrovirals, *HCV* hepatitis C virus, *ADR* adverse drug reaction, *SOF* sofosbuvir, *DCV* daclatasvir, *LDV* ledipasvir, *OBV/PTV/r* ombitasvir/paritaprevir/ritonavir, *RBV* ribavirin.

^a^Chi-Squared Test.

^b^Fisher’s Exact Test.

After excluding ADRs with calculated IC less than 1 and PRR less than 2 (also excluding ICs and PRRs 95% CI values including the null value; 1 for PRRs and 0 for IC), and N_obs_ less than 3, the number of ADR signals for disproportionate reporting (SDRs) was 35 for sofosbuvir/daclatasvir, 11 for ombitasvir/paritaprevir/ritonavir, 7 for sofosbuvir/ribavirin and 4 for sofosbuvir/ledipasvir.

Concerning SOF/LDV, the SDRs developed were thrombocytopenia (IC 2.81, 95% CrI 0.738–4.01), off label use (IC 2.63, 95% CrI 1.76–3.23), HCV relapse (IC 2.12, 95% CrI 0.7–3.03) and anaemia (IC 1.87, 95% CrI 0.312–2.86) (Table 2).

**Table 2:** Signals of Disproportionate Reporting (SDRs) for the ADRs Reported with DAA Regimens Prescribed for Egyptian Patients with Chronic HCV in (2925) Reports from (2013) to (2021).

DAA	ADR	*N*_obs_^a^	*N*_*e*xp_^b^	*N*_reaction_^c^	IC (95% CrI)	PRR (95% CI)
SOF/DCV	MPGN	24	1	25	4.03 (3.35–4.51)	716 (96.9–5290)
(± RBV)	Blood bilirubin increased	97	7	208	3.70 (3.36–3.94)	26.1 (19.9–34.1)
	Hepatitis C reactivation	308	24	731	3.65 (3.47–3.79)	21.7 (18.9–25.0)
	Hepatic cirrhosis	17	1	40	3.54 (2.73–4.11)	22.1 (11.8–41.2)
	Hepatocellular carcinoma	16	1	37	3.46 (2.62–4.04)	22.7 (11.9–43.5)
	Menstrual disorder	26	2	61	3.41 (2.75–3.87)	22.2 (13.4–36.7)
	Gastritis	137	13	414	3.35 (3.07–3.55)	14.8 (12.1–18.0)
	Flatulence	70	7	230	3.23 (2.84–3.52)	13.1 (9.89–17.2)
	Splenomegaly	21	2	66	3.10 (2.37–3.62)	13.9 (8.31–23.3)
	Hepatic encephalopathy	11	1	38	2.94 (1.91–3.63)	12.2 (6.04–24.5)
	Treatment failure	41	5	157	2.92 (2.40–3.29)	10.5 (7.41–15.0)
	Gastrointestinal motility disorder	3	0	9	2.81 (0.74–4.01)	14.9 (3.73–59.6)
	Hepatitis B reactivation	10	1	19	2.81 (1.73–3.53)	33.1 (13.5–81.5)
	Plicated tongue	3	0	5	2.81 (0.74–4.01)	44.8 (7.48–268)
	Prothrombin time prolonged	3	0	9	2.81 (0.74–4.01)	14.9 (3.73–59.6)
	Conjunctivitis	8	1	25	2.50 (1.29–3.31)	14.0 (6.07–32.5)
	Thrombocytopenia	102	18	541	2.47 (2.14–2.71)	6.93 (5.61–8.56)
	Dizziness	335	63	1936	2.40 (2.22–2.53)	6.24 (5.60–6.96)
	Anaemia	202	42	1289	2.25 (2.02–2.42)	5.54 (4.80–6.40)
	Acute kidney injury	11	2	61	2.20 (1.18–2.90)	6.56 (3.42–12.6)
	Arthralgia	93	20	631	2.19 (1.85–2.44)	5.16 (4.16–6.40)
	Abdominal pain	207	47	1453	2.13 (1.90–2.29)	4.96 (4.31–5.70)
	Headache	375	93	2881	2.01 (1.84–2.13)	4.46 (4.04–4.93)
	Fatigue	309	95	2941	1.70 (1.51–1.83)	3.50 (3.14–3.91)
	Nervousness	20	6	189	1.66 (0.91–2.18)	3.53 (2.23–5.60)
	Decreased appetite	54	17	539	1.64 (1.19–1.96)	3.32 (2.52–4.38)
	Hepatic failure	16	5	164	1.58 (0.74–2.17)	3.23 (1.93–5.39)
	Drug interaction	17	6	177	1.43 (0.61–2.00)	3.17 (1.93–5.22)
	Pain	138	51	1565	1.43 (1.15–1.63)	2.89 (2.44–3.42)
	Somnolence	75	29	894	1.36 (0.97–1.63)	2.73 (2.17–3.45)
	Pruritus	99	39	1211	1.33 (1.00–1.57)	2.66 (2.17–3.25)
	Constipation	52	21	661	1.29 (0.83–1.62)	2.55 (1.93–3.37)
	Abdominal pain upper	73	30	929	1.27 (0.88–1.55)	2.54 (2.01–3.22)
	Vision blurred	61	26	805	1.21 (0.79–1.52)	2.45 (1.89–3.16)
	Leukopenia	15	7	227	1.05 (0.18–1.65)	2.11 (1.25–3.56)
OBV/PTV/r	Drug interaction	25	0	177	5.67 (5.00–6.14)	60.1 (40.5–89.3)
(± RBV)	Blood bilirubin increased	22	1	208	3.91 (3.19–4.41)	43.2 (28.5–65.5)
	Pruritus	35	3	1211	3.34 (2.78–3.74)	10.9 (8.05–14.7)
	Gastritis	13	1	414	3.17 (2.23–3.81)	11.8 (6.96–20.2)
	Decreased appetite	11	1	539	2.94 (1.91–3.63)	7.61 (4.27–13.6)
	Anaemia	32	4	1289	2.85 (2.26–3.27)	9.30 (6.77–12.8)
	Haemoglobin decreased	7	1	349	2.32 (1.02–3.17)	7.48 (3.59–15.6)
	Renal impairment	6	1	246	2.12 (0.70–3.03)	9.14 (4.12–20.3)
	Fatigue	34	8	2941	2.02 (1.45–2.43)	4.27 (3.16–5.79)
	Arthralgia	7	2	631	1.58 (0.28–2.44)	4.10 (1.98–8.50)
	Dizziness	15	5	1936	1.49 (0.62–2.10)	2.85 (1.76–4.64)
SOF/RBV	Anaemia	385	14	1289	4.73 (4.56–4.85)	39.3 (35.9–43.1)
	Ascites	22	1	87	3.91 (3.19–4.41)	31.3 (19.4–50.4)
	Hepatitis C reactivation	109	8	731	3.69 (3.37–3.92)	16.2 (13.4–19.6)
	Coma hepatic	5	0	44	3.46 (1.90–4.44)	11.8 (4.68–30.0)
	Hepatic encephalopathy	4	0	38	3.17 (1.40–4.25)	10.9 (3.87–30.5)
	Hepatitis B reactivation	3	0	17	2.81 (0.74–4.01)	19.8 (5.70–68.7)
	Jaundice	7	1	105	2.32 (1.02–3.17)	6.60 (3.08–14.2)
SOF/LDV	Thrombocytopenia	3	0	541	2.81 (0.74–4.01)	6.84 (2.28–20.6)
(± RBV)	Off-label use	15	2	2269	2.63 (1.76–3.23)	8.17 (5.33–12.5)
	Hepatitis C reactivation	6	1	731	2.12 (0.70–3.03)	10.2 (4.77–21.6)
	Anaemia	5	1	1289	1.87 (0.31–2.86)	4.79 (2.08–11.0)

*ADR* adverse drug reaction, *DAA* direct-acting antiviral, *HCV* hepatitis C virus, *IC* information component, *PRR* proportional reporting ratio, *CI* confidence interval, *CrI* credibility interval, *DCV/SOF* daclatasvir/sofosbuvir, *OBV/PTV/r* ombitasvir/paritaprevir/ritonavir, *SOF/RBV* sofosbuvir/ribavirin, *SOF/LDV* sofosbuvir/ledipasvir, *RBV* ribavirin, *MPGN* membranoproliferative glomerulonephritis.

^a^*N*_observed_: the actual number of case reports for the drug-reaction combination [1].

^b^*N*_expected_: the number of case reports expected for the drug-reaction combination [1].

^c^*N*_reaction_: the number of case reports for the ADR irrespective of drug [1].

For OBV/PTV/r, from the highest SDRs were drug interaction (IC 5.67, 95% CrI 5–6.14), anaemia (IC 2.85, 95% CrI 2.26–3.27), and renal impairment (IC 2.12, 95% CrI 0.7–3.03) (Table 2).

Concerning SOF/DCV, the highest SDRs were hepatic and blood disorders, from the highest SDRs were increased levels of blood bilirubin (IC 3.7, 95% CrI 3.36–3.94), HCV relapse (IC 3.65, 95% CrI 3.47–3.79), hepatocellular carcinoma (IC 3.46, 95% CrI 2.62–4.04) and splenomegaly (IC 3.1, 95% CrI 2.37–3.62) (Table 2).

Similarly, for SOF/RBV, the highest SDRs were hepatic and blood disorders. The highest signal being anaemia (IC 4.73, 95% CrI 4.56–4.85), followed by ascites (IC 3.91, 95% CrI 3.19–4.41), HCV relapse (IC 3.69, 95% CrI 3.37–3.92) and HBV reactivation (IC 2.81, 95% CrI 0.738–4.01) (Table 2).

From the results of descriptive and disproportionality analyses, estimates of safety and effectiveness -per IC, severity, fatality and seriousness indices- for the four DAA regimens are summarized in (Table 3).

**Table 3:** Comparative Safety and Effectiveness Per the IC Values, Severity, Fatality and Seriousness Indices.

Parameter	ADR	OBV/PTV/r	SOF/DCV	SOF/LDV	SOF/RBV
		IC (95% CrI)			
Effectiveness	HCV relapse	1.58 (− 2.20 to 3.27)	3.65* (3.47 to 3.79)	2.12* (0.70 to 3.03)	3.69* (3.37 to 3.92)
	Cirrhosis	–	3.54* (2.73 to 4.11)	2.32 (− 0.27 to 3.71)	1.58 (− 2.20 to 3.27)
	ALF	–	1.58* (0.74 to 2.17)	–	− 0.74 (− 4.52 to 0.95)
	HE	1.30 (− 2.50 to 2.89)	2.94* (1.91 to 3.63)	2.32 (− 0.27 to 3.71)	3.17* (1.40 to 4.25)
	Ascites	–	0.36 (− 1.40 to 1.44)	2.32 (− 0.27 to 3.71)	3.91* (3.19 to 4.41)
Safety	Renal impairment	2.12* (0.70 to 3.03)	2.20* (1.18 to 2.90)	2.32 (− 0.27 to 3.71)	− 0.74 (− 4.52 to 0.95)
	HBV reactivation	–	2.81* (1.73 to 3.53)	–	2.81* (0.74 to 4.01)
	Anaemia	2.85* (2.26 to 3.27)	2.25* (2.02 to 2.42)	1.87* (0.31 to 2.86)	4.73* (4.56 to 4.85)
	HCC	–	3.46* (2.62 to 4.04)	–	1.58 (− 2.20 to 3.27)
Severity index		0.53%	1.67%	3.9%	4.02%
Seriousness index		9.5%	26%	35.3%	85.7%
Fatal outcome		0%	0.9%	2%	1.2%

Each of the severity, seriousness and fatality indices were calculated individually for each regimen as percent of the number of ICSRs involving the incident to the total number of ICSRs reported. All values of descriptives are represented in (Table 1).

*ADR* adverse drug reaction, *ALF* acute liver failure, *CrI* credibility interval, *HBV* hepatitis B virus, *HCC* hepatocellular carcinoma, *HCV* hepatitis C virus, *HE* hepatic encephalopathy, *IC* information component, *OBV/PTV/r* ombitasvir/paritaprevir/ritonavir, *SOF/DCV* sofosbuvir/daclatasvir, *SOF/LDV* sofosbuvir/ledipasvir, *SOF/RBV* sofosbuvir/ribavirin.

‘*’ indicates that the estimate was statistically significant and met the criteria of signals of disproportionate (ICs greater than 1, IC 95% credibility interval (95% CrI) lower-bound greater than 0, and, the number of reports was ≥ 3).

‘─’ indicates that the ADR was not reported in any of the ICSRs.

Comparing to the ‘Legacy treatment’ reference group,^1^ logistic regression analysis^2^ (Table 4) showed that the risk of renal impairment was highest with SOF/LDV (adjusted ROR 8.13, 95% CI 1.18–34.38) and OBV/PTV/r (ROR 3.67, 95% CI 1.26–9.44). For anaemia, the highest risk was with OBV/PTV/r (ROR 8.97, 95% CI 4.91–17.29) and SOF/DCV (ROR 1.42, 95% CI 1.12–1.81). For HCV/HBV reactivation, the highest risk was with the legacy treatment (SOF) while the lowest was with OBV/PTV/r (ROR 0.04, 95% CI 0.01–0.11), the same was for the risk of hepatic complications was highest with the legacy treatment (SOF) while the lowest with OBV/PTV/r (OR 0.03, 95% CI 0.01–0.10).

**Table 4:** Logistic Regression Analysis to Identify the Direct-Acting Antivirals Association with Selected Serious Events While Adjusting for Age, Gender, Pre-existing Cirrhosis and Ribavirin Use.

Predictors	Renal impairment	Anaemia	HCV/HBV Reactivation	Hepatic Complications
	OR (95% CI)	OR (95% CI)	OR (95% CI)	OR (95% CI)
(Intercept)	0.00*** (0.00–0.01)	1.25 (0.69–2.26)	1.27 (0.69–2.34)	1.16 (0.63–2.13)
Age (years)	1.05** (1.02–1.08)	0.98*** (0.97–0.99)	1.01* (1.00–1.02)	1.01* (1.00–1.02)
Gender (Male vs. Female):	0.94 (0.50–1.76)	0.26*** (0.20–0.33)	4.72*** (3.70–6.05)	5.35*** (4.18–6.89)
Cirrhosis (Present vs Absent):	1.03 (0.06–5.17)	0.72 (0.26–1.69)	0.58 (0.28–1.21)	1.37 (0.64–3.14)
Ribavirin included in regimen:	0.95 (0.49–1.87)	3.06*** (2.38–3.96)	0.15*** (0.11–0.19)	0.15*** (0.11–0.19)
Treatment (vs. Sofosbuvir):				
DCV/SOF	1.05 (0.52–2.07)	1.42** (1.12–1.81)	0.64*** (0.50–0.82)	0.63*** (0.49–0.81)
OBV/PTV/r	3.67* (1.26–9.44)	8.97*** (4.91–17.29)	0.04*** (0.01–0.11)	0.03*** (0.01–0.10)
LDV/SOF	8.13* (1.18–34.38)	1.22 (0.25–4.47)	0.17** (0.04–0.58)	0.23 * (0.06–0.79)

*OR* odds ratio, *HBV* hepatitis B virus, *HCV* hepatitis C virus, *OBV/PTV/r* ombitasvir/paritaprevir/ritonavir, *SOF/DCV* sofosbuvir/daclatasvir, *SOF/LDV* sofosbuvir/ledipasvir.

*P < 0.05, **P < 0.01, ***P < 0.001.

Concerning the impact of age on the selected ADRs, increasing age was significantly associated with a higher risk of renal impairment (OR 1.05, 95% CI 1.02–1.08), HCV/HBV reactivation (OR 1.01, 95% CI 1–1.02), and hepatic complications (OR 1.01, 95% CI 1–1.02).

Regarding the gender, male patients were at a lower risk of anaemia (OR 0.26, 95% CI 0.2–0.33). However, male patients were at higher risk of HCV/HBV reactivation (OR 4.72, 95% CI 3.7–6.05), and hepatic complications (OR 5.35, 95% CI 4.18–6.89).

Finally, ribavirin inclusion independently increased the risk of anaemia (OR 3.06, 95% CI 2.38–3.96). However, ribavirin was associated with a lower risk of HCV/HBV reactivation and hepatic complications (OR 0.15, 95% CI 0.11–0.19).

## Discussion

In this study, we found unusual ADR signals associated with certain DAAs, particularly the signals of renal impairment and anaemia with OBV/PTV/r that was independent of ribavirin use as revealed by the logistic regression analysis, and the disproportionate reporting signal of HCC with SOF/DCV. The reported signals were exceedingly high, clinically relevant, and unexpected, given the normal use of DAAs and the natural progression of HCV infection. These findings mandate further research to confirm the exposure-effect relationship and to further validate the clinical relevance.

Comparing the results of descriptive and disproportionality analyses among the four treatment regimens revealed that OBV/PTV/r regimen was superior to the other DAAs concerning treatment effectiveness and compensated disease. Besides, OBV/PTV/r had the least reported serious, severe and fatal outcomes, indicating a favourable risk-benefit, despite the reported high signals of anaemia and renal impairment which mandate close monitoring of renal and hematologic parameters.

On the other hand, the highest percentage of severe outcomes, the highest signal of HCV relapse and associated hepatic complications and positive signals of anaemia and HBV reactivation were reported with SOF/RBV. Our conclusions regarding the risk-benefit evaluation are valid for the time of this cross-sectional study and may require an update in subsequent analysis.

This is the first pharmacovigilance study that highlighted the safety of DAAs in HCV-infected Egyptian patients using data from the WHO VigiBase, which includes a considerable number of reports. We performed a disproportionality analysis using Bayesian and frequentist signal indicators (ICs and PRRs), and multivariate logistic regression analysis to adjust for clinically relevant covariates. Our approach differs from Cazacu et al. (2020) and Hayes et al. (2021), who were only concerned with descriptives to evaluate DAAs' safety using VigiBase [13, 32].

Most of the ADRs were deemed mild per the Hartwig's severity assessment scale (49.5%). Serious ADRs comprised 38.7%. The most commonly reported ADRs were anaemia (21.33%), HCV relapse (14.53%), headache (14.02%), fatigue (12.99%), dizziness (12.24%) and pruritis (4.85%). Similarly, fatigue, headache, and pruritis were among the most common ADRs reported by Cazacu et al. (2020) and Lashen et al. (2018) [32, 33], while Hashmi et al. (2021), Gheorghe et al. (2017) and Ghoneem et al. (2021) [12, 14, 34] reported anaemia, fatigue, and headache.

Our results regarding the most commonly reported ADRs were not much different from those reported in clinical trials on DAA regimens in concern; Kowdley et al. reported fatigue [35], headache and anaemia, Zeuzem et al. reported fatigue, headache and pruritis [36], Afdhal et al. and Nelson et al. reported fatigue and headache [37, 38], while Chayama et al. reported nasopharyngitis and headache [39].

Concerning OBV/PTV/r, the disproportionality analysis showed that the highest signal for OBV/PTV/r was for drug–drug interactions (DDIs), supported by the work of Cazacu et al. (2020), who highlighted potential DDIs in 1.9% and major DDIs in 20.4% of all ICSRs included in their analysis; Amlodipine was the medicine most frequently involved in the ICSRs with reported major DDIs, followed by furosemide, metformin, omeprazole and digoxin [32].

OBV/PTV/r, however, was also associated with serious and potentially fatal ADRs including anaemia and renal impairment. The observed number of reports of renal impairment were twofold higher than the expected number of reports (IC 2.12, 95% CrI 0.7–3.03) (PRR: 9.14, 95% CI 4.12–20.3), these results are in line with those results of Ashram et al. (2021), who reported high PRRs for renal side effects with OBV (5.53), PTV (4.88), and ritonavir (2.84) [40].

The logistic regression analysis also showed that OBV/PTV/r was independently associated with nearly fourfold risk of renal impairment. In addition, it showed that advancing age was associated with increased risk of renal impairment (ROR 1.05, 95% CI 1.02–1.08). These previously mentioned findings are in agreement with Cazacu et al. (2020), who reported an age-dependent risk of renal impairment with OBV/PTV/r [32].

Concerning anaemia, Gheorgh et al. (2017) reported anaemia as the most frequent ADR observed with OBV/PTV/r (33.5%) [34]. In the present work, OBV/PTV/r was associated with nearly ninefold risk on anaemia, which was independent of ribavirin use (ROR 8.97, 95% CI 4.91–17.29). The strikingly high ROR for OBV/PTV/r induced anaemia calls for further studies to confirm a clinical correlation, if any. Despite the heightened risk of anaemia and renal impairment, OBV/PTV/r was superior to all other studied DAA combinations in terms of lower risk of HCV/HBV reactivation and progression to hepatic encephalopathy and HCC and being the regimen reported with the lowest severity index (0.53%) and fatal outcomes (0%).

HCV relapse was a common SDR reported with SOF/RBV (IC 3.69, 95% CrI 3.37–3.92), SOF/DCV (IC 3.65, 95% CrI 3.47–3.79) and SOF/LDV (IC 2.12, 95% CrI 0.7–3.03). However, Lashen et al. (2018) did not report any relapses with SOF/DCV and relapse in only 1.2% of patients with HCV GT‐4 on SOF/LDV treatment [33]. In another study by Abergel et al. (2016), relapse was reported in 6.8% of the HCV GT‐4 patients on SOF/LDV [41]. Possible explanation of the reported relapse rates with SOF/DCV regimen in our study is the non-inclusion of RBV in 63.5% of the cases, considering that adding RBV to DAA regimens may reduce relapse rates as noted by Ahmed et al. (2018) [42], which supports our finding that RBV was indeed associated with a lower risk of HCV/HBV reactivation and hepatic complications (OR 0.15, 95% CI 0.11–0.19).

A SDR of hepatocellular carcinoma (HCC) was observed with SOF/DCV (IC 3.46, 95% CrI 2.62–4.04), which was comparable with Lashen et al. (2018) findings, who reported discontinuation in 8 patients (1.3%) due to HCC development while on treatment with SOF/DCV [33]. HCC was also reported in two previous studies on patients treated with SOF/DCV; Hassany et al. (2020) reported HCC development in 22 (6.7%) patients who achieved SVR and 5 (23.8%) patients who failed to achieve SVR concluding that the occurrence of HCC is significantly higher in patients who failed to achieve SVR and that treatment response decreases the risk of HCC occurrence [6]. However, Aziz et al. (2019) reported that 10 (3.33%) of their study patients developed HCC, from which 5 patients achieved SVR, suggesting that the frequency of HCC following SOF/DCV regimen is independent of achieving SVR [43].

On contrary, other studies negates the correlation between the DAA treatment and HCC; Lin et al. (2020) concluded from their study that DAA therapy did not only increase the probability of HCC recurrence in patients who received curative treatment for HCC but it also improved the survival outcome of those patients [44]. Moreover, in a large cohort of North American patients by Singal et al. (2019), it was found that there was no significant difference in the HCC recurrence patterns between the DAA-treated patients and the untreated ones supporting that DAA therapy was not associated with the increased HCC recurrence [45].

Regarding the impact of gender on the risk of HCC, the logistic regression analysis showed that male patients were at higher risk of hepatic complications including HCC (OR 5.35, 95% CI 4.18–6.89), which supports the work of Cazacu et al. (2020), who highlighted the relationship between gender and neoplasms reporting higher percentage in male patients [32].

Our study had certain limitations. First, the total number of patients included in the analysis is the total number of patients who reported ADRs only, not the actual number of patients who received DAAs in Egypt, this is due to the issue of underreporting [18]. Second, the study did not include the role of drug interactions in the incidence of the reported ADRs. Third, Among the four studied DAA combinations, there were disparities in the frequency of reports which may had affected our comparisons; the highest proportion of 2032 reports were with SOF/DCV, and the lowest proportion of only 51 reports was with SOF/LDV. Finally, the findings resulting from the use of pharmacovigilance data and signal detection methods have to be explored for clinical validation as ADR reporting is a passive process that cannot provide a measurement of the true risk associated with the drug [13, 18, 46].

## Conclusion

OBV/PTV/r was superior to other regimens in terms of lower risk of HCV/HBV reactivation and hepatic complications and being the regimen with the lowest severity index (0.53%), seriousness (9.5%) and fatal outcomes (0%). However, OBV/PTV/r correlated with fourfold higher risk of renal impairment and ninefold higher risk of anaemia than SOF based regimens, which calls for further population-based studies for clinical validation.

The DAA combination SOF/RBV reported the highest severity index (4.02%) and seriousness (85.7%). besides, the highest signal of HCV relapse among the four regimens was reported with SOF/RBV which is possibly related to the efficacy of regimen.

## Footnotes

1 Legacy treatment group includes SOF, SOF + RBV and SOF + IFN regimens.

2 The total number of reports eligible for logistic regression analysis was 1837 reports.
